# The disutility of compartmental model forecasts during the COVID-19 pandemic

**DOI:** 10.3389/fepid.2024.1389617

**Published:** 2024-06-20

**Authors:** Tarini Sudhakar, Ashna Bhansali, John Walkington, David Puelz

**Affiliations:** Salem Center for Policy, Department of Finance, & Department of Information, Risk, and Operations Management, Austin, TX, United States

**Keywords:** compartmental models, SIR, SEIR, pandemic surveillance, COVID-19

## Abstract

During the COVID-19 pandemic, several forecasting models were released to predict the spread of the virus along variables vital for public health policymaking. Of these, the susceptible–infected–recovered (SIR) compartmental model was the most common. In this paper, we investigated the forecasting performance of The University of Texas COVID-19 Modeling Consortium SIR model. We considered the following daily outcomes: hospitalizations, ICU patients, and deaths. We evaluated the overall forecasting performance, highlighted some stark forecast biases, and considered forecast errors conditional on different pandemic regimes. We found that this model tends to *overforecast* over the longer horizons and when there is a surge in viral spread. We bolstered these findings by linking them to faults with the SIR framework itself.

## 1 Introduction

Forecasts of the spread of COVID-19 in the United States and across the world have played a significant role in informing policymakers. Employing different mathematical approaches, these forecasting models provide predictions that inform critical decisions such as healthcare administration, allocation of medical supplies, and business and school closures. Some models even attempted to estimate the impact of current and future policies on human behavior, COVID-19 transmission, and vaccinations (1–3). As the COVID-19 pandemic recedes, there is a significant opportunity to take stake of the predictions compartmental models generated. Indeed, this was the first major pandemic in the modern “computational era,” where simulations were cheap and accessible to scientists. Researchers quickly fit these models and advised world leaders on pandemic surveillance and decision-making. This paper presents a rigorous study of these compartmental model predictions, including the magnitude of forecasting mistakes and a novel investigation of the systematic bias induced by this technology.

For the Austin-Round Rock metropolitan statistical area, forecasts given by the University of Texas (UT) COVID-19 Modeling Consortium shaped COVID-19 policies to a significant extent such as the introduction of staged lockdowns (4). These had serious repercussions on the overall economy, spanning the closure of small businesses to setbacks in K-12 students due to hybrid or online education. Statewide, Texas is estimated to have suffered a GDP loss of $106 million and job losses of 1.2 million (5). Texas also suffered unprecedented setbacks in student achievement in reading and mathematics due to the mass transition to remote learning. The Texas Education Agency cites a roughly 3-month setback in educational attainment relative to a pre-COVID baseline. From 2019 to 2021, the percentage of Texas students performing at or above their grade level in math decreased from 50% to 35% (6). While we have moved on from such strict measures for now, some questions remain unanswered. Did we base these policies on well-sourced data? In the future, can we still rely on these forecasting models to aid our decision-making?

Research from the US COVID-19 Forecast Hub (1) demonstrated that standalone models tend to generate large prediction errors, especially when forecasting over a long time horizon. A main contribution of this paper is to utilize a rich set of predictions coupled with realized data to investigate the bias of these errors. The current state of literature reports error summary statistics, like mean absolute or squared deviation, as well as coverage of prediction intervals, as in Fox et al. (7). A related approach relies on visualizing the prediction errors (residuals), as in Kumar Ghosh et al. (8), where they assert that “residuals of the fit are randomly distributed, which imply that the fitness of the data with the model is overall good.” The lack of rigorous focus on forecast errors is a major gap in the epidemiological literature, especially since the sole task of these models is prediction. What remains understudied and obfuscated in these statistics and visualizations are the time dependency and systematic biases in these errors. Specifically, this paper probes the following set of questions: Are prediction errors from compartmental models systematically biased in positive or negative directions? If so, to what degree, why, and are these errors related to time? We analyzed an informative dataset of realized hospitalizations, deaths, and ICU patients coupled with forecasts of these three outcomes from the UT COVID-19 Modeling Consortium. The UT Austin model is an advanced compartmental model with frequent, long-horizon forecasts, making it an important one to study. Given its impact on COVID-19 policymaking, we investigate the UT model’s forecasting performance in light of realized data; discover that forecast errors are biased, predictable, and dependent on the pandemic regime; and propose paths forward and alternative approaches.

## 2 Overview of compartmental models

Most researchers adopted the susceptible–infected–recovered (SIR) model and its modifications to forecast COVID-19 metrics due to its simplicity and prevalence among academics and training of epidemiologists (9). The SIR modeling framework falls under the category of compartmental models that date back to the seminal work of Ross (10).

In these models, individuals within a closed population are separated into mutually exclusive groups, or compartments, based on their disease status. At any given time, each individual is considered to be in one compartment but can move to another compartment based on the model parameters. As per the model’s assumptions, a susceptible individual will become infected by coming into contact with an infected individual. The individual during the infected period is assumed to be contagious. After this, the individual advances to a non-contagious state, known as recovery. Recovery may also be death or effective isolation (11). The subgroups modeled by this framework are given by the following notation:

• *S* is the fraction of susceptible individuals who are able to contract the disease,
• *I* is the fraction of infectious individuals who can transmit the disease, and
• *R* is the fraction of recovered individuals who have become immune.

Once these groups are defined, the trajectory of this system, known as the classical SIR model where the subgroups are the state variables, is given by the following set of differential equations:

(1) $$\begin{array}{ccc} \frac{\partial S}{\partial t}\; & =\; & -\beta IS,\;\\ \frac{\partial I}{\partial t}\; & =\; & \beta IS-\gamma I,\;\\ \frac{\partial R}{\partial t}\; & =\; & \gamma I.\; \end{array}$$

State variables S, I, and R are all functions of time. Model parameters β and γ are the rate of transmission and recovery, respectively. The first equation in Equation 1 models the fraction of people susceptible to the virus at a certain point, given the transmission rate and the fraction of infected individuals. The second equation in Equation 1 models the instantaneous fraction of infected individuals using susceptible individuals and the rate of recovery. The third equation in Equation 1 models the instantaneous fraction of recovered individuals using the rate of recovery.

Modified SIR models, such as susceptible–exposed–infected–recovered (SEIR), require a more complex set of equations and parameters. Due to the presence of little information and lack of reliable data at the beginning of the COVID-19 pandemic, many researchers relied on the classical SIR implementation (12). Nonetheless, the setup for this modified model formulation is an extension by the SIR framework by one additional compartment, denoted “exposed.” The state variable for this new compartment is E (13). Here, we assume that there are equal birth and death rates μ, α is the mean latency period for the virus, γ is the mean infectious period, the rate of transmission as before is β, and recovered individuals do not contract the disease again. This admits the following set of equations:

$$\frac{\partial S}{\partial t}=\mu -\beta IS-\mu S,$$

$$\frac{\partial E}{\partial t}=\beta IS-(\mu +\alpha )E,$$

$$\frac{\partial I}{\partial t}=\alpha E-(\mu +\gamma )I.$$

Since each variable is defined as a fraction of the entire population, we calculate *R* from the equation S+E+I+R=1.

Similar to the SIR model, this system describes the population evolution of the virus in terms of intuitive parameters that summarize its interaction with humans, like ease of transmission, incubation period, and length of recovery. Conveniently, it is easy to extend this framework to higher-dimensional systems. The number of compartments can be extended, new parameters can be added, and time variation in parameters can be incorporated, as shown in Girardi and Gaetan (14) and Spannaus et al. (15).

As a final practical note, a useful metric derived from this system is called the basic reproduction number $R_{0}$, which represents the average number of infections generated from an infected individual within a susceptible population. During the COVID-19 outbreak, this was a closely watched and debated number since it describes the evolution of the virus itself.

$$R_{0}=\frac{\beta \alpha }{\left(\mu +\alpha )(\mu +\gamma \right)}.$$

The reproduction number (also called effective reproductive number) is a time-varying version of the basic reproduction number and denotes the evolving transmissibility of the disease. See Dharmaratne et al. (16) for a detailed discussion of these two important epidemiological concepts.

In the following subsections, we provide two examples of compartmental model use during the COVID-19 pandemic. The examples build on each other and demonstrate the flexibility of compartmental modeling. First, we summarize the work done by the Institute for Health Metrics. Second, we describe the additional features incorporated into the model developed by the UT COVID-19 Modeling Consortium. UT’s model forecasts are the focus of this paper.

### 2.1 Example 1: Institute for Health Metrics and Evaluation

The Institute for Health Metrics and Evaluation (IHME) COVID-19 Forecasting Team located at the University of Washington created multiple models over the course of the pandemic. The initial model garnered much press and attention and used statistical curve-fitting to estimate hospital bed utilization, ICU admissions, ventilator use, and deaths from 25 March 2020 to 29 April 2020 (17).

In this model, the IHME team took a different approach to model death rates compared with the classical compartmental framework. They critiqued SEIR models for their assumption of random mixing between all individuals in the population because, under that assumption, millions of COVID-19-related deaths were predicted very early in the United States. Random mixing does not account for behavioral changes and government-mandated social distancing measures. Instead, the IHME team modeled actual COVID-19 death rates since they would indicate virus transmission and fatality rates. According to the authors, deaths were also more accurately reported than cases, especially in limited testing areas as those would allocate tests for severely ill patients first. They also assumed hospitalization and related services to be highly correlated with deaths.

Their next model took on a hybrid approach, estimating revised death rates and then fitting an SEIR model until 26 May 2020 (18, 19). The third model replaced curve-fitting with spline-fitting for the relationship between log cumulative deaths and log cumulative cases while retaining the SEIR model estimation (18, 20). When tested against other forecasting models for predictive accuracy, this third IHME model exhibited the best performance (out of seven models) (18).

### 2.2 Example 2: the UT COVID-19 Modeling Consortium

The UT COVID-19 Modeling Consortium developed a highly publicized and widely used model to predict trends in key pandemic variables, including hospitalizations, deaths, and ICU patients, in the Austin-Round Rock metropolitan statistical area (21).^1^ Motivated by the IHME approach, the UT team developed an alternative curve-fitting method for forecasting COVID-19 outcome variables. While the underlying technology remained a compartmental model, the team layered in other sources of hierarchical information and complexity.

1. Mobile phone data to capture social distancing measures: To capture the effect of changing social distancing measures for each US state on individual-level mobility, the model used local data from mobile phone GPS traces from SafeGraph.^2^
2. A correction for the underestimation of uncertainty in the IHME forecasts: Using deaths as an example outcome, the IHME model estimated cumulative death rates using a least-squares-like procedure on the log scale and calculated confidence intervals based on large-sample statistical theory. For this method to produce valid uncertainty measures, consecutive model errors should be independent of each other. However, this assumption is violated in the IHME fitting procedure since today’s cumulative death rate includes yesterday’s death rate and an increment. This implies that these two death rates must be correlated. The UT model corrected this by fitting daily death rates using a mixed-effects negative-binomial generalized linear model, accounting for heteroskedasticity and correlation.

In the following sections, we investigate the forecasts generated from the UT model and describe telling features of the forecast errors.

## 3 Methodology

We now focus exclusively on the forecasts generated from the UT COVID-19 Modeling Consortium. We first describe our methodology for analyzing the errors and then turn to our results. Our analysis began with forecast error computation and visualization. We then constructed a new variable defining “pandemic regime,” which describes whether viral spread is surging or waning. This allowed us to condition data on regime status and fit regression models that describe prediction error as a function of the regime status. In detail, our analysis of the UT forecasting model for hospitalizations, ICU patients, and deaths followed these steps:

1. Visualizing forecasts against realized data: We plotted realized data with forecasts generated by the UT model. While we have daily realized data on hospitalizations, ICU patients, and deaths, we did not have the same for forecasts as the model was not updated daily and had a different schedule for each outcome variable.
2. Mapping forecasting errors from 1 to 20 days ahead: We computed the forecasting errors out to 20 days ahead, showing the median forecasting error and interquartile range (IQR) of the error distribution. The forecast error for a given number of days out (denoted d) at a particular time point t is calculated as follows:
  (2) $$E_{t+d,t}=F_{t+d,t}-A_{t+d},$$
  where
  • $E_{t+d,t}$ is the forecast error for the forecast made at time t for time t+d,
  • $A_{t+d}$ is the actual (realized) value at time t+d, and
  • $F_{t+d,t}$ is the predicted value at time t for time t+d.
3. Mapping forecasting errors, conditional on whether the virus is spreading or waning: We computed the forecasting errors from 1 to 20 days out, conditional on whether there is a surge in the viral spread.
  For each outcome of interest, we defined rising hospitalizations/ICU patients/deaths as surging and falling hospitalizations/ICU patients/deaths as waning. We constructed this new variable in two steps. First, we computed the daily percent change in the outcome of interest. Second, to smooth out noise in the realized data, we took a 14-day moving average of the realized outcome’s percent change. Our final definition of a rising (falling) point in time t is a day that has a 14-day average percent change of greater (less) than zero.
  Using the realized data for a given outcome $A_{t}$, the percent change is given by
  $${\dot{A}}_{t}=\frac{A_{t}-A_{t-1}}{A_{t-1}}\times 100.$$
  The smoothed data (over a 14-day rolling window) is then defined in the following way:
  $${\dot{A}}_{t}=\frac{1}{14}\sum_{k=t-14+1}^{t}{\dot{A}}_{k}.$$
  With ${\dot{A}}_{t}$ in hand, we can now define a new indicator variable that summarizes the state of the pandemic according to the smoothed growth or abatement of the outcome of interest. The new variable $I_{t}^{\text{rising}}$ describes whether or not time t is a “rising” or “falling” regime in the following way:
  (3) $$I_{t}^{\mathrm{rising}}=1({\dot{A}}_{t}>0)=\{\begin{array}{cc} 1\; & \mathrm{if}\;{\dot{A}}_{t}>0,\;\\ 0\; & \mathrm{if}\;{\dot{A}}_{t}\leq 0,\; \end{array}$$
  where 1(⋅) is the indicator function.

Our final methodological step was to empirically investigate the forecast errors through a regression model. We were interested in testing whether the errors are related to the pandemic regime, so we specified the following Gaussian linear regression model:

(4) $$E_{t+d,t}=\eta +\theta \times I_{t}^{\text{rising}}+\epsilon_{t},\;\epsilon_{t}∼N(0,\sigma^{2})$$

where η is the intercept, θ is the slope of the regression model, and d={1,5,10,20} denotes the forward time horizons we considered. Moreover, we fitted 12 models, 4 forward time horizons for each of hospitalizations, deaths, and ICU capacity. The slope θ has an intuitive interpretation as the difference in average prediction error between rising and falling pandemic regimes. Therefore, the results of θ’s inference are crucial to understanding the relationship between forecast mistakes and the “state of the pandemic.” The model is fit using ordinary least squares (OLS), and we report point estimates and standard errors to gauge statistical significance in the next section.

## 4 Results

**Figure 1:**
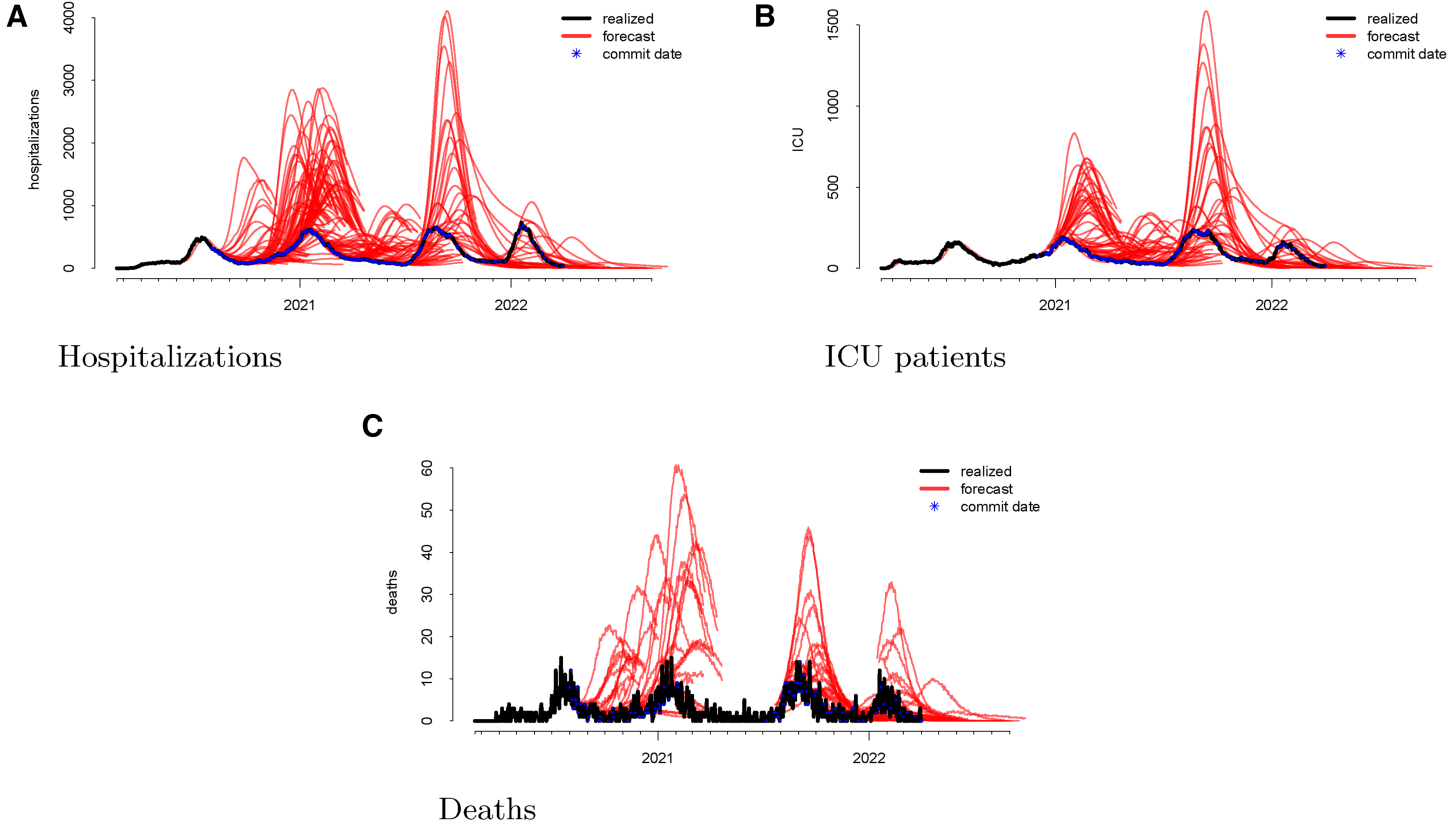
Visualizations of the raw data for hospitalizations (panel A), ICU patients (panel B), and deaths (panel C). The realized data are displayed in black, the forecasts are given in red, and the commit dates (the points at which the forecasts are created) are given in blue along the realized path.

Following the methodological steps outlined in the previous section, we now investigate each of the three outcomes forecasted by the UT model. In the data visualizations, each figure panel corresponds to one of hospitalizations, deaths, or ICU patients. Figure 1 displays the raw data. The realized data, in black, are daily and starts on 19 February 2020 and ends on 1 April 2022. The forecasts, in red, start on 1 August 2020 and end on 1 April 2022. Although we have daily realized data, the model was not updated daily. Instead, there are 246 forecast dates, denoted “commit dates” in the plot and shown in blue. These are the dates when the entire model with new forecasts is updated, i.e., when each of the red forecasts begin.

**Figure 2:**
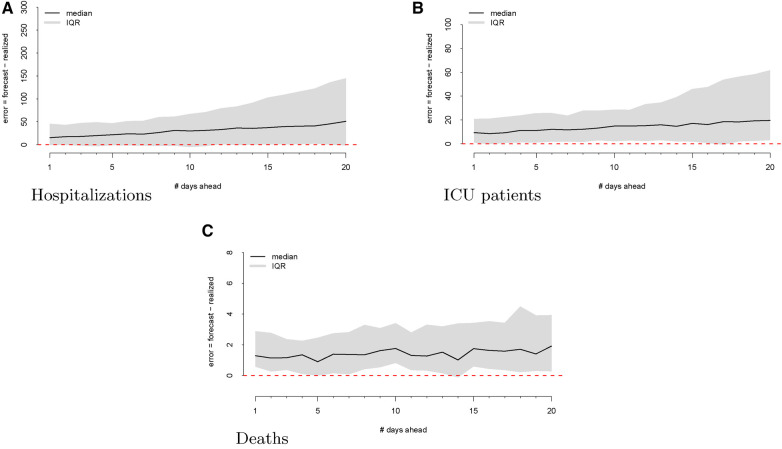
Forecasting errors from 1 to 20 days ahead for hospitalizations (panel A), ICU patients (panel B), and deaths (panel C). The median forecast error for each period ahead is displayed by the black line, and 25th to 75th quantiles of the error distribution are represented by the gray region.

As a first step, we computed the forecasting errors out to 20 days ahead, as displayed in Figure 2. The median forecasting error is shown in black, and the IQR of the error distribution is the shaded region. An immediately visible feature is the upward bias of the error distribution, even via the median error, which is robust to egregiously large forecasts. This implies that the model systematically *overforecasts* hospitalizations.

We next considered visualizing the errors conditional on different stages of the pandemic. Our goal was to answer the question: Does the model systematically *over- or underforecast* when the virus is spreading more rapidly or is receding? In other words, is the model more likely to make forecasting errors during variant surges or when the viral spread is waning? To answer this question, we defined rising hospitalizations as surging and falling hospitalizations as waning. To smooth out noise in the realized data, we computed the percent change in the realized hospitalizations and smoothed with a 14-day rolling window. Our final definition of a rising (falling) regime is a day that has a 14-day average percent change of greater (less) than zero.

**Figure 3:**
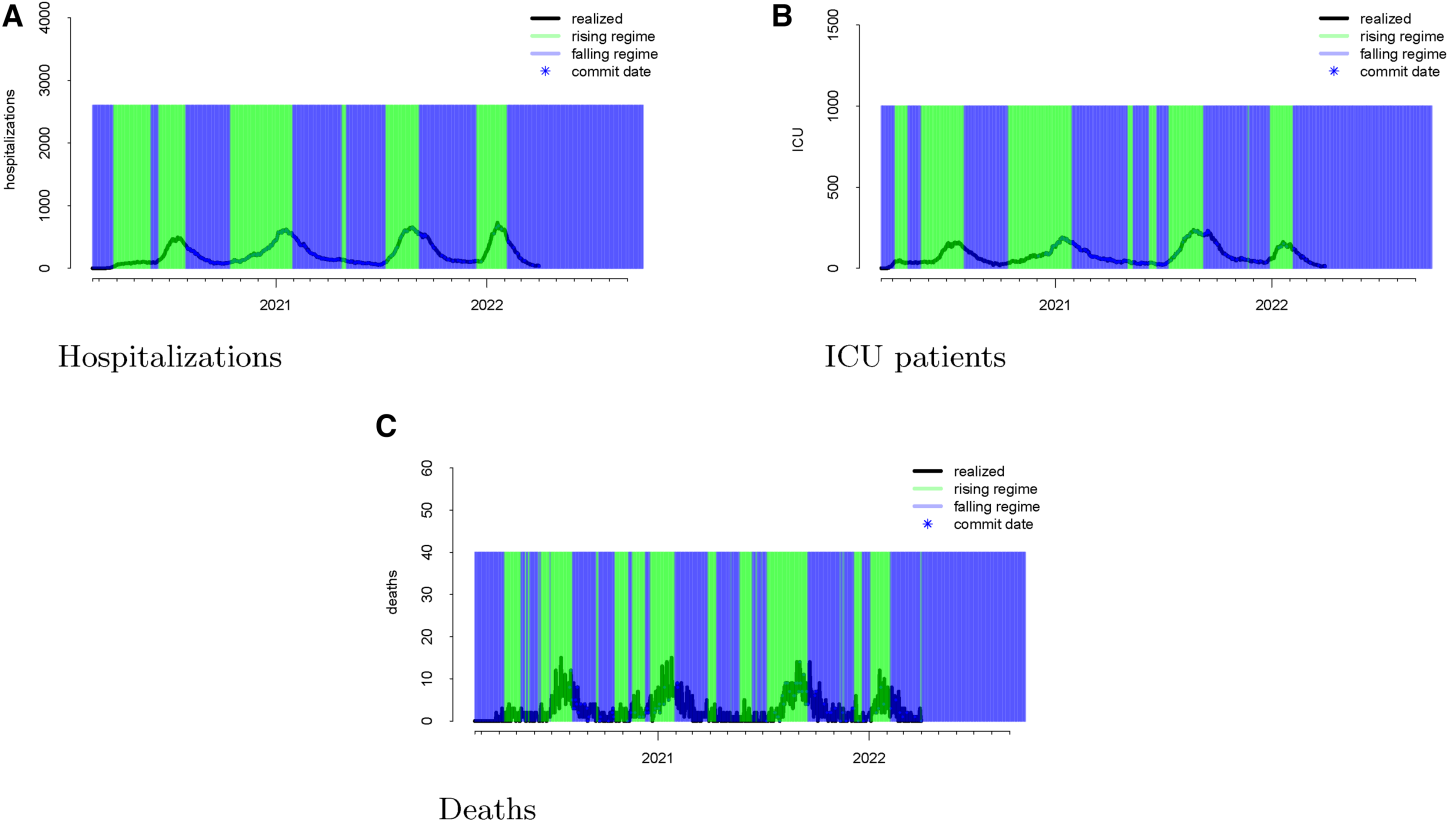
Realized data with “rising” and “falling” regimes overlayed in green and blue, respectively for hospitalizations (panel A), ICU patients (panel B), and deaths (panel C). “Rising” is defined as a day when the 14-day moving average percent change in hospitalizations exceeds zero. “Falling” is defined as a day when the 14-day moving average percent change in hospitalizations is below zero.

**Figure 4:**
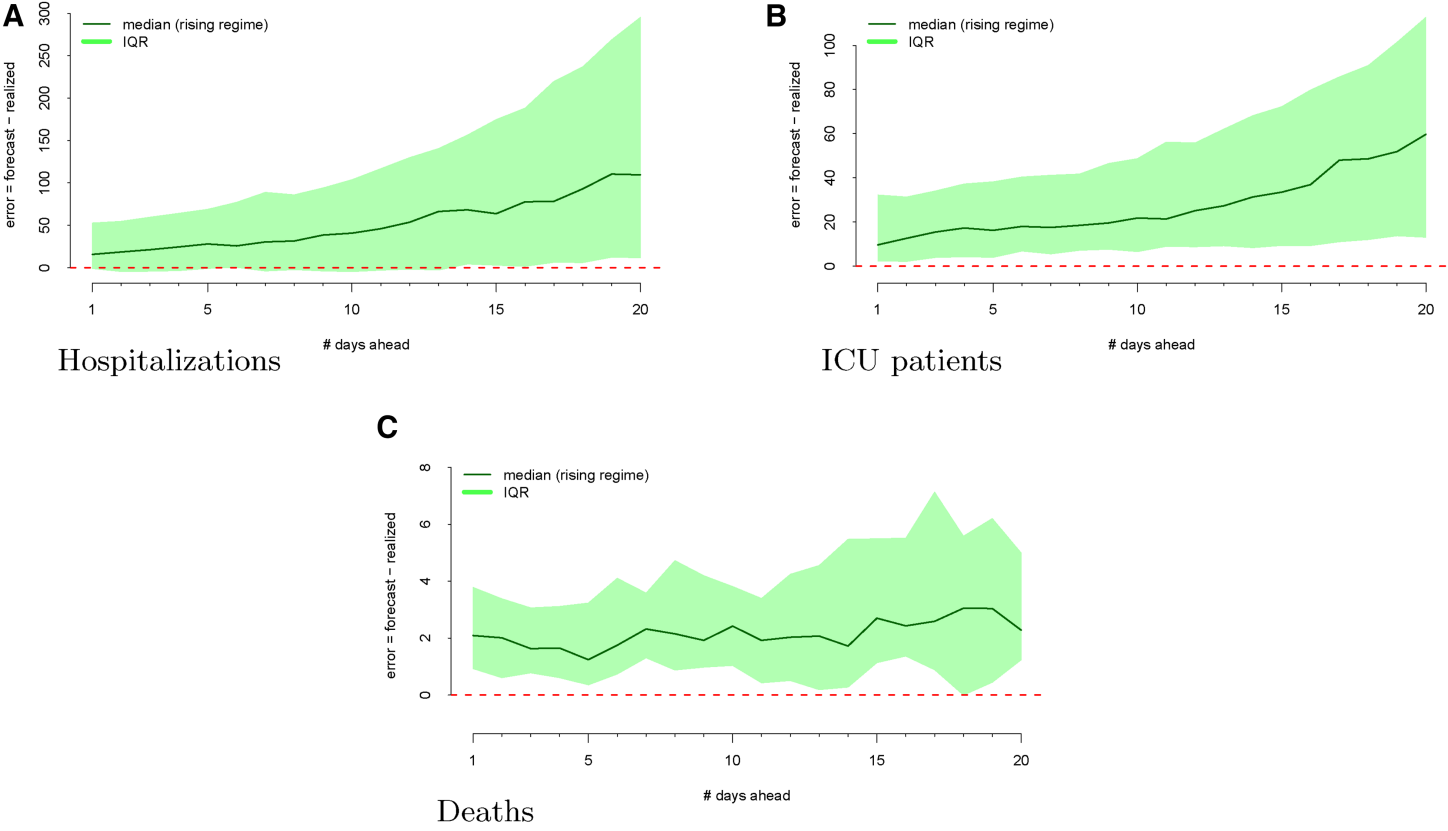
Forecasting errors from 1 to 20 days ahead, conditional on **forecasting in a rising regime** for hospitalizations (panel A), ICU patients (panel B), and deaths (panel C). The median forecast error for each period ahead is displayed by the bold line, and 25th to 75th quantiles of the error distribution are represented by the shaded region.

**Figure 5:**
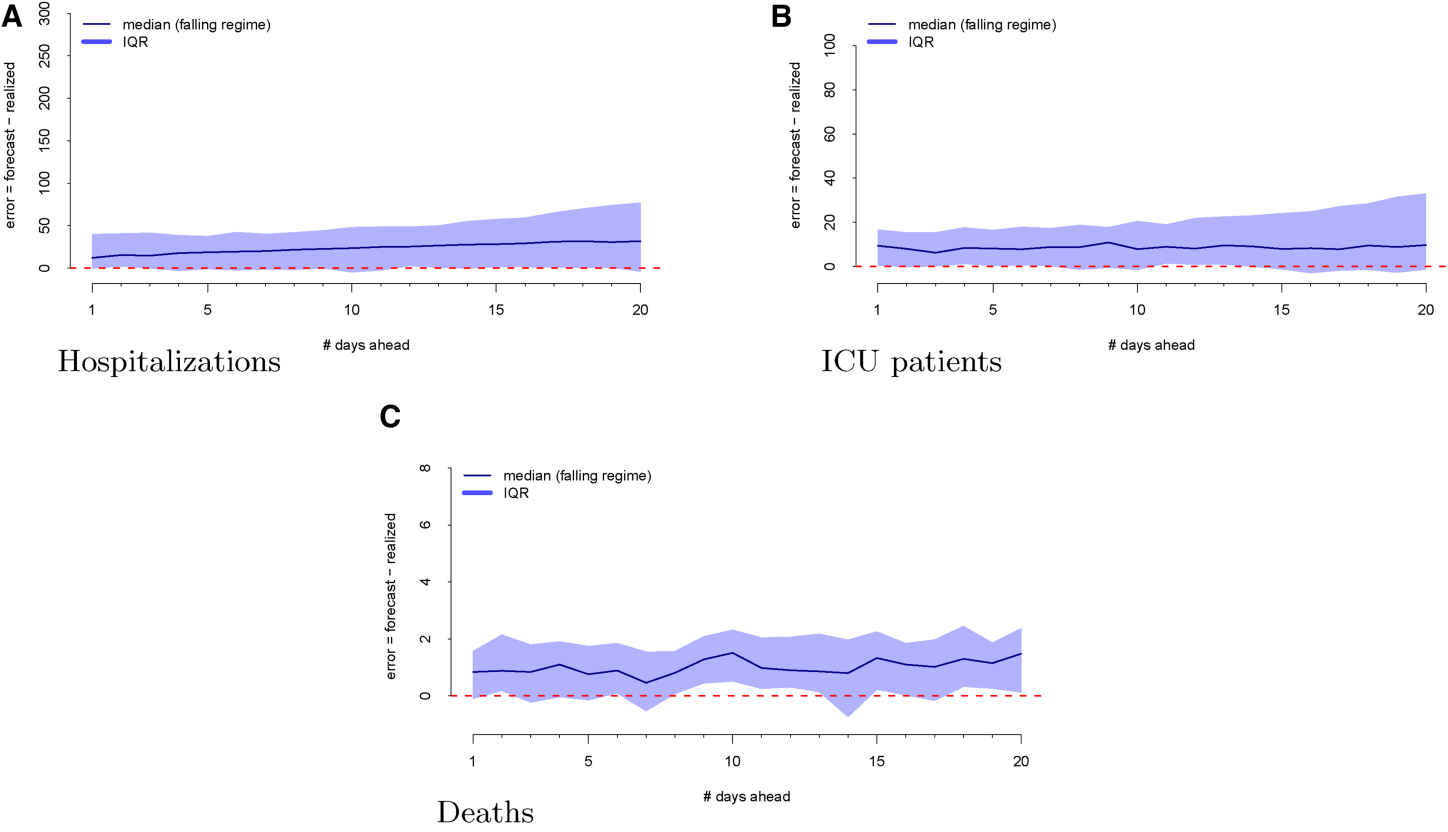
Forecasting errors from 1 to 20 days ahead, conditional on **forecasting in a falling regime** for hospitalizations (panel A), ICU patients (panel B), and deaths (panel C). The median forecast error for each period ahead is displayed by the bold line, and 25th to 75th quantiles of the error distribution are represented by the shaded region.

The regimes as defined above are displayed in Figure 3. The forecast errors conditional on these regimes (analogous to Figure 2) are displayed in Figures 4 (rising) and 5 (falling). When looking at these figures, an interesting feature emerges. The model makes large positive mistakes during surges (and with larger variance, Figure 4) compared to when the viral spread is waning (Figure 5) and hospitalizations are falling.

We then investigate the number of ICU patients and its corresponding forecasts. Figure 1 displays the raw data vs. the forecasts at each commit date. Similar to our work on hospitalizations, we calculated the forecasting error up to 20 days ahead, as shown in Figure 2. There is a clear upward bias, especially for predictions generated 15–20 days ahead. This implies again that the model systematically *overforecasts* the number of ICU patients.

We mapped the errors to the rising and falling regimes defined by the realized ICU admission data. As defined in Section 2.2, rising ICU admissions correspond to a surge in the viral spread, and falling ICU admissions denote waning viral spread. Figure 3 visualizes these rising and falling regimes. We see a distinct trend emerge in the rising regime. Errors in the model are larger and more positive when ICU admissions are surging compared to when the ICU admissions are falling.

Finally, we looked at the daily COVID-19 deaths. Figure 1 shows the model’s forecasts (in red), along with the actual values (in black). This is similar to the plots shown above for hospitalizations and ICU admissions, where the realized values are daily but aperiodic, and the forecasts lines begin on their respective commit dates.

Figure 2 shows the forecasting errors of the model computed with forecasts up to 20 days ahead. Similar to the previous error plots, the black line shows the median forecasting error, while the IQR is shaded in gray. Interestingly, deaths exhibit a weaker upward bias compared to ICU and hospitalization errors.

Once again, we display the realized data during the “rising” and “falling” regimes in Figure 3. To assess whether the model has different forecasting errors during a “rising” regime or a “falling” one, we plotted the errors conditioned on the regime when these values were forecast.

Figure 4 shows the model’s errors during a “rising” regime, whereas Figure 5 shows the model’s errors during a “falling” regime. Unlike with hospitalization and ICU forecasts, the errors are similar and more attenuated in both regimes.

### 4.1 Empirical analysis of model errors: are the errors predictable?

We conclude our analysis of the SIR model errors by fitting several regression models relating the forecast error to the viral regime. Specifically, we regressed the forecast error at 1, 5, 10, and 20 days ahead on the indicator variable of whether or not the forecast date was a “rising regime,” as defined in Equation 3. The unit of analysis for these regressions is the commit date for each outcome since this is when the forecasts were generated; the outcome is the forecasting error d days ahead, as given by Equation 2. The regression model Equation 4 was fitted using OLS for 12 outcomes: 4 forward time horizons for each of hospitalization, deaths, and ICU patients.

Tables 1–3 correspond to the regression fits for hospitalization, ICU, and death forecast errors, respectively. Each table shows the intercept and coefficient estimates for each regression in a column, as well as standard errors in parentheses and *p*-values noted in asterisks. Each coefficient on the indicator variable represents the average difference in error between rising and falling regime forecasts.

**Table 1:** Hospitalization forecast errors regressed on rising regime indicators.

Outcome	1 day ahead	5 days ahead	10 days ahead	20 days ahead
Intercept	20.24 (3.29)***	20.64 (4.47)***	24.96 (7.18)***	42.72 (14.99)**
I (rising regime)	8.80 (4.87)	18.75 (6.61)**	37.58 (10.62)***	125.99 (22.12)***
R^2^	0.01	0.03	0.05	0.12
Adj. R^2^	0.01	0.03	0.05	0.11

*** *p* < 0.001; **P < 0.01; *P < 0.05.

**Table 2:** ICU forecast errors regressed on rising regime indicators.

Outcome	1 day ahead	5 days ahead	10 days ahead	20 days ahead
Intercept	11.97 (2.08)***	10.89 (2.47)***	11.04 (3.31)**	15.29 (6.70)*
I (rising regime)	4.55 (3.09)	10.91 (3.67)**	18.82 (4.92)***	58.50 (9.93)***
R^2^	0.01	0.05	0.09	0.18
Adj. R^2^	0.01	0.05	0.08	0.18

*** *p* < 0.001; **P < 0.01; *P < 0.05.

**Table 3:** Death forecast errors regressed on rising regime indicators.

Outcome	1 day ahead	5 days ahead	10 days ahead	20 days ahead
Intercept	0.95 (0.42)*	0.77 (0.51)	1.50 (0.46)**	1.73 (0.71)*
I (rising regime)	1.82 (0.59)**	1.03 (0.72)	1.26 (0.64)	2.22 (0.98)*
R^2^	0.10	0.02	0.04	0.06
Adj. R^2^	0.09	0.01	0.03	0.05

*** *p* < 0.001; **P < 0.01; *P < 0.05.

Across all outcomes, there are strongly significant differences in forecast errors depending on the viral regime, especially for forecasts 10 and 20 days ahead. All estimated indicator coefficients are positive. For example, the hospitalization model errors are 125.99 (Table 1) greater in rising regimes than in falling regimes. This predictability of errors is shocking since forecast errors should be random.

## 5 Discussion: modeling challenges

The SIR model fails to properly model certain aspects of disease spread. Melikechi et al. (22) pointed out that over the last century, many have modified the SIR model to incorporate different compartments for various subpopulations or added new terms that identify unique pathogen transmissions. However, adding too many features may lead to overfitting when making inferences of parameters early on in an epidemic (23). At the beginning of an epidemic or pandemic, there is a risk of noisy observations. Melikechi et al. (22) raised the concept of *practical identifiability*, which refers to the ability to “discern different parameter values based on noisy observations.” Most SIR models employ Monte Carlo simulations, where the model is simulated with predetermined parameters. Noise is added to the simulated data, and then, a fitting procedure is used on the noisy data. With increase in the magnitude of noise, Monte Carlo parameter estimates often display large difference in values, leading to huge uncertainty in the parameters and unreliable inferences.

Lemoine (24) dissected missteps of a popular SEIR model built by Flaxman et al. (25), in which they analyzed the effect of non-pharmaceutical interventions on deaths due to the virus. Flaxman et al. (25) used partial pooling of information between countries, with both individual and shared effects on the time-varying reproduction number. According to them, pooling allowed for more information to be used and helped overcome country-specific idiosyncrasies in the data to enable more timely estimates. The authors argued that government lockdowns were the interventions that made a bulk of the impact on controlling the viral spread. Lemoine, however, pointed out that the overall effect of government interventions on deaths in Sweden was similar to that in other countries. Why is this important? Sweden was the one country in the analysis that did not have a full lockdown. When he reproduced their analysis, Lemoine found that the country-specific effect for Sweden that the model ignored was almost as large as the effect of a full lockdown, a feature that the authors failed to present in their findings. Based on this, Lemoine argued that such SIR models have been unable to deliver useful inputs for policymakers. However, more importantly, epidemiologists have failed to acknowledge this fact, by not ascribing the failure of their models to the right causes.

There are three major critiques for the SIR models: assumption of homogeneous mixing, assumption of closed population, and latency period of infection.

### 5.1 Homogeneous mixing

SIR models assume random mixing between all individuals in a given population. Based on this assumption, each individual has the same amount of contact as everyone else. Such a model would not be able to account for a higher contact rate at hospitals or a lower contact rate for quarantined individuals (26). There is a lack of adequate inclusion of individual behavioral and social influence in SIR models. Infectious disease epidemics have a substantial social aspect and public health implication. Homogeneous mixing assumes an equal probability of transmission between two people regardless of their age or location (27). This can fail to take into account age-dependent or location-dependent risks. We need to include varying degrees of interventions such as social distancing, stay-at-home, and shelter-in-place orders at different times and across different regions. The assumption of homogeneous mixing of *S* with *I* state individuals in the SIR model is therefore invalid during COVID-19.

This flaw of SIR models may explain why the errors tend to be so high with the UT Austin model. While homogeneous mixing can be helpful for projecting the number of cases, it can lead to large errors both in the early stages of the epidemic and in calculating the final epidemic size (27). Homogeneous mixing often overestimates the epidemic’s size, and can lead to more interventions than needed. While the UT model accounts for different age groups and risk factors, it still models disease transmission through an SIR framework where the base assumption remains homogeneous mixing. That is, all individuals within each group would have the same susceptibility to infection, and all individuals within each infection status compartment would have the same infectiousness.

Even with more spatially explicit metapopulation models, homogeneous mixing at smaller scales, such as within a state, county, or city, is still questionable, as some people can stay home and some are essential. In addition, the regional variability of individual sentiment and behavior, for example, whether to obey or enforce these orders, is essential to determine to predict the trajectory of the COVID-19 pandemic, but it is generally not included in the SIR models (28).

Assumption of homogeneous mixing can lead to overestimating health service needs by not accounting for behavioral changes and government-mandated actions. In Wuhan, strict social distancing was instituted on 23 January 2020, and by the time new infections reached 1 or fewer a day (15 March 2020), less than 0.5% of the population was infected. At the time, SIR models generally suggested that 25%–70% of the population to be infected (17).

Since most SIR models consider a single $R_{0}$ value, they miss unexpected social behavior changes and are unable to follow the alterations. For instance, social gatherings have a great impact on disease spread. A religious event in Malaysia, held from 27 February to 3 March, was supposed to be the source of viral spread in India and Pakistan (12, 29).

Chen et al. (28) argued that since SIR models are formulated at the population level, we face an important discrepancy between patient-level data and population-level modeling. Exposed (*E*) and infectious (*I*) compartments characterize the disease spread at the population level, ignoring individual clinical variations in patients. Due to its broadness, the *E* compartment assumes that everyone exposed is unable to infect others and the *I* compartment does not account for varying levels of severity among patients such as asymptomatic, mild, and severe stages. Given these assumptions, SIR models cannot pin down and quantify the impact of superspreaders who can lead to a disproportionately large number of new cases. Superspreading can be due to individual clinical characteristics such as supershedding of virus or behavioral aspects like supercontacting. Neither are addressed well by SIR models built at a population level. This flaw in the SIR models may also explain some of the inaccuracy in the UT model, as not accounting for patient-level differences may cause them to overlook individual behaviors and *overforecast*.

### 5.2 Closed population

The focus of SIR models is often placed on the estimation of the basic reproduction number $R_{0}$ (30). However, what should be addressed is the assumption of a closed population in SIR models. Most regions do not follow complete isolation, making them vulnerable to changes in the neighboring communities. SIR models also consider recovered individuals to be immunized. This assumption contrasts with the possibility of the reactivation of the virus or reinfection of previously infected individuals (12). Similarly, it does not account for asymptomatic individuals. With the closed population assumption, Ding et al. (31) argued that standard SIR models miss out on the fact that presymptomatic and asymptomatic cases can spread the disease between populations through travel. Researchers have addressed this at multiple levels: within state, country, and even globally.

Studies such as those by Kucharski et al. and Wu et al. (32, 33) estimated cases in Wuhan, China, by considering the movement people in and out of the city. Kucharski et al. (32) did so by assuming that once exposed, a part of the population would travel internationally. To account for international travelers, they used the number of outbound travelers (assuming 3,300 per day before travel restrictions were imposed on 23 January 2023, and zero afterward), relative connectivity of different countries, and relative probability of reporting a case outside Wuhan vs. within Wuhan and internationally.

Wu et al. (33) first inferred the $R_{0}$, the basic reproduction rate of the virus, of COVID-19 and the outbreak size in Wuhan from 1 December 2019 to 25 January 2020, on the basis of confirmed cases exported from Wuhan to cities outside of mainland China, where symptom onset date was reported to range from 25 December 2019 to 19 January 2020. They also forecasted the spread of COVID-19 within and outside Mainland China, taking into account public health interventions and outbound travelers by air, train, and road during the Spring Festival. While they assumed that travel behavior was not affected by the disease and, therefore, international case exportation occurred according to a non-homogeneous process, their work still addresses mobility across cities and countries for modeling COVID-19.

Chinazzi et al. (34) used a global epidemic and mobility model (GLEAM) to model the international spread of COVID-19, considering varying transmissibility and air traffic reductions. The model uses a metapopulation network approach with real-world data, where the world is divided into subpopulations centered around major transportation hubs such as airports. The subpopulations are connected to each other by individuals traveling daily through them. COVID-19 transmission within each subpopulation is modeled through a susceptible–latent–infectious–recovered compartmental framework.

Ding et al. (31) differentiated their study by incorporating granular changes in air traffic and simulate varying travel restrictions. They focused on data from Canada, which showed a large number of flights going to and fro the country despite travel restrictions and reduced air traffic. They proposed a modified SIR model that considers a dynamic flight network, by estimating imported cases using air traffic volume and positive testing rates. Their model operates in an “open population setting,” where people are free to travel in and out of the population.

Not accounting for a lack of closed population leads to poor estimation of forecasts. Depending on the assumptions and circumstances of the model, this assumption can lead to both underestimation and overestimation. For instance, Chowell and Nishiura (35) revealed that in the case of the Ebola virus, variations in the $R_{0}$ number in an SEIR model were due to different assumptions regarding the international or domestic spread of the virus and the lack of high-quality data.

In our analysis of the UT Austin model, we captured the model’s systematic tendency to *overforecast*. UT’s initial $R_{0}$ values in 2020 were “best guesses,” as seen in the study by Wand et al. (36). They assumed $R_{0}$ to be 2.2 but did not provide a clear source for how they arrived at this number.^3^ Tec et al. (37) estimated $R_{0}$ using the basic infectiousness of the disease, the number of people susceptible to infection, and the impact of social distancing, mask wearing, and other measures to slow transmission. One potential reason why the UT model *overforecasts* could be due to their social distancing data. They used mobility trends from SafeGraph data and regressed the transmission rate of the virus on the first two principal components derived from a principal component analysis (PCA) on eight independent mobility variables, such as home dwell time and visits to universities, bars, grocery stores, museums and parks, medical facilities, schools, and restaurants. If the PCA components did not appropriately capture the variation in the mobility, such as inter-Austin movement, then the transmission rate would lead to a poor estimation of the viral spread.

### 5.3 Latency period of infection

The SIR model also does not incorporate the latent period between when an individual is exposed to a disease and when that individual becomes infected and contagious. This is because the only categories in the model are susceptible, infectious, and recovered. The S(E)IR model tries to account for this parameter by creating a category for people who are exposed but not yet contagious. However, even S(E)IR models are oversimplified, and they will need to model other time-dependent factors, such as the introduction of community mitigation strategies (11).

Changes in model parameters *E*, *I*, or *R* at time *t* is dependent on a fraction of *E* and *I* at time *t*. As per Liu (38), this means that after being exposed to the virus on a particular day, an individual may become contagious or recover on that same day. However, in reality, an exposed individual will become infectious only after a latent period and recover after an infectious period (38). This timing issue with the compartmental models can cause the forecasts to be inaccurate, especially for further days out.

SEIR models account for this latency period using an “exposed” compartment, but even this model feature is too structured and simplistic. If the latency period is not calibrated precisely, the mode may *over/underforecast*. A study using COVID-19 data in Tennessee found that the “optimal” latency period is 2.40 days, which is close to the mean latency period of 2.52 days, which was estimated from the data of seven countries (39). If UT Austin’s SEIR model did not correctly model this latency period, this could explain some of the model’s errors.

## 6 Conclusion

Given the wide use of compartmental models to describe the transmission dynamics of COVID-19 and other diseases, we must carefully consider their limitations when using them to inform public health interventions. In particular, homogeneity assumptions underlying these models do not accurately reflect heterogeneity of the population, and estimates of key parameters such as $R_{0}$ are often noisy and unreliable. In addition, these models do not account for the impact of non-pharmaceutical interventions on disease transmission or capture the complex interactions between the virus, people, and the environment.

Historically, compartmental models were used as descriptive tools instead of real-time models for prediction and decision-making. This change in “use-case” partially explains their significant failure. Chen et al. (28) pointed out that in the initial papers describing the SIR approach, the model was applied after the epidemic had ended. However, SIR models have little room for new evidence without modifying the model structure and estimation of $R_{0}$. In addition, it was difficult to model the initial part of COVID-19 spread using SIR models, compared to other models, since we had limited or no information on aspects such as asymptomatic transmission, superspreaders, and unreported cases. Moreover, constructing a long-term forecasting model is a major challenge because of the lack of data. The SIR model has a tendency to underestimate peak infection rates and substantially overestimate the persistence of the epidemic after the peak has passed (40).

Intuitively, prediction accuracy can be increased by synthesizing the forecasts of many unique models. This represents one important area of future research to improve the usefulness of compartmental models. Cramer et al. (1) found that an ensemble model provides more accurate short-term forecasts of hospital and ICU admissions than the individual models alone. The ensemble model also has a lower prediction error and better calibration than the individual models, suggesting that it could be more effective in real-time decision-making for healthcare systems.

In this paper, we investigated predictive failures of the compartmental model framework and its derivative models. While our empirical analysis utilized forecasts from the model constructed at UT Austin, our conclusions apply to all compartmental models generated from sets of differential equations. The epidemiological community should scrutinize this framework and promote new and innovative techniques that go beyond differential equations and adopt solutions from economics, statistics, machine learning, and beyond. A pandemic forecasting model can always be marketed as unique, but if it relies on differential equations and compartmental modeling, this structural bias will outweigh most novel modifications. We hope this manuscript contributes to a rebirth of research in epidemiological modeling so the best techniques can be elevated and utilized in future pandemics.

## Data Availability

The original contributions presented in the study are included in the article/Supplementary Material; further inquiries can be directed to the corresponding authors.

## Footnotes

^1^The data are available on the UT team’s GitHub directory. These forecasts are also visually available to the public on their website.

^2^More information on SafeGraph can be found here.

^3^The authors linked a source, but it is not open access.
